# Pedicled myofascial temporalis flap for closure of large maxillary defects after medication-related osteonecrosis of the jaws. A case series

**DOI:** 10.1007/s10006-026-01532-w

**Published:** 2026-02-26

**Authors:** Sanne Werner Moeller Andersen, Liezl Dawson, Iben Poulsen, Simon Storgaard Jensen, Thomas Kofod

**Affiliations:** 1 Department of Oral and Maxillofacial Surgery, Rigshospitalet, Copenhagen University Hospital, Blegdamsvej 9, 2100 Copenhagen, Denmark; 2 Research Area Oral Surgery, Section for Oral Biology and Immunopathology, Institute of Odontology, Faculty of Health and Medical Sciences, University of Copenhagen, Copenhagen, Denmark

**Keywords:** Bisphosphonate-associated osteonecrosis of the jaws, Surgical flaps, Bone density conservation agents, Temporal muscle, Maxillary disease

## Abstract

**Background:**

Medication-related osteonecrosis of the jaws (MRONJ) located in the maxilla may lead to challenging oro-antral and oro-nasal defects too extensive to be predictably closed with local soft tissue flaps. The use of a pedicled myofascial temporalis flap (PMTF) is, however, well-established for closing large maxillary defects following ablative craniomaxillofacial surgery.

**Objectives:**

The purpose of the present study was to evaluate the use of PMTF for maxillary defect closure in patients with stage 3 MRONJ.

**Methods:**

A retrospective cohort study was conducted based on data from the Copenhagen ONJ cohort from 1 January 2005 to 31 December 2024. The inclusion criteria were consecutive patients with extensive maxillary defects after surgical treatment of MRONJ and reconstruction with PMTF.

**Results:**

Seven patients met the inclusion criteria (three with cancer, two with osteoporosis, and two with both conditions). All defects were successfully closed using the PMTF. Healing was uneventful in all patients at the recipient site, with complete mucosal closure achieved. Postoperative pain was reduced in all cases. One patient developed a late donor-site complication requiring removal of the temporal implant; no flap-related failures occurred.

**Conclusions:**

The removal of necrotic bone combined with radical sinusotomy and closure of the defect with PMTF appears to be a feasible approach for managing extensive maxillary MRONJ lesions, with favourable outcomes observed in this limited cohort.

## Introduction

Medication-related osteonecrosis of the jaws (MRONJ) is a serious and potentially debilitating adverse effect associated with antiresorptive treatment (AR) and other oncologic therapies. The condition is defined and staged according to the criteria of the American Association of Oral and Maxillofacial Surgeons (AAOMS) [1–3]. Although MRONJ most frequently affects the mandible, maxillary involvement accounts for approximately one-quarter of cases and is often associated with extension into the maxillary sinus or nasal cavity, thereby meeting the criteria for stage 3 [2, 4–6].

Maxillary MRONJ poses distinct diagnostic and therapeutic challenges. Due to its anatomical concealment and frequent absence of early symptoms, maxillary disease is often diagnosed at an advanced stage [7, 8]. If left untreated, infection may spread to adjacent anatomical structures, resulting in serious complications such as nasal septal abscess [9], orbital cellulitis [10], or necrotic lesions extending to the skull base [11]. Advanced maxillary MRONJ frequently necessitates extensive surgical resection, including partial or hemi-maxillectomy, which may involve removal of teeth, the alveolar process, hard palate, and portions of the maxillary sinus or nasal walls which may lead to changed facial appearance, impaired masticatory function, impaired speech and swallowing, and nasal fluid leakage [12], ultimately affecting Health-Related Quality of Life (HRQoL) [13].

Reconstruction of large oro-antral and oro-nasal defects remains challenging, particularly in patients with impaired wound healing, comorbidities, and ongoing or previous AR. Several reconstructive options have been described, including the use of an obturator prosthesis [12, 14, 15], the buccal fat pad [13, 16], the nasolabial flap [17], the pedicled myofascial temporalis flap (PMTF) [18], and the microvascular free flap [19, 20]. However, no consensus exists regarding the optimal reconstructive approach for stage 3 maxillary MRONJ [2, 21]. Treatment strategies range from conservative management to extensive surgical resection with or without reconstruction, depending on defect size, patient health status, and anticipated functional outcomes. Nonetheless, there is general agreement that treatment should aim to control infection, alleviate pain, halt disease progression, and improve HRQoL [2].

The PMTF is a well-established reconstructive option in craniofacial and maxillofacial surgery, particularly for closure of large maxillary defects following oncologic or traumatic resections [18, 22]. Its robust vascularity, proximity to the maxilla, and ability to provide substantial soft-tissue bulk make it an attractive option for complex intraoral defects. Despite these advantages, the use of PMTF for the reconstruction of maxillary defects secondary to MRONJ has not previously been reported.

Temporal depression is a common consequence of PMTF harvest unless reconstructed [23]. Various materials and techniques have been described for temporal fossa reconstruction, including porous high-density polyethylene (PHDPE) implants [24], polymethyl methacrylate (PMMA) [25], titanium implants (TI) [26], Mersilene mesh (MM) [27], autologous fat transplantation (lipofilling) [28], and polyetheretherketone (PEEK) [29] polyetherketoneketone (PEKK) [30]. Among these, PHDPE (Medpor®) has demonstrated favourable long-term outcomes, with tissue ingrowth, resistance to infection, and a lower complication rate compared with PMMA [23, 31–34]. More recently, advances in virtual surgical planning and PSI fabrication may further improve donor-site reconstruction outcomes [30, 35].

To the authors' knowledge, no published data exist on the closure of maxillary stage 3 MRONJ-related defects with PMTF. The primary aim of this study was to evaluate clinical outcomes following reconstruction of extensive maxillary MRONJ defects using the PMTF. The secondary aim was to assess donor-site outcomes following temporal fossa reconstruction with PHDPE implants.

## Material and methods

### Study design and setting

This retrospective cohort study was conducted using data from the Copenhagen ONJ Cohort, which includes all consecutive patients diagnosed with MRONJ and treated at the Department of Oral and Maxillofacial Surgery, Copenhagen University Hospital, Denmark, between 1 January 2005 and 31 December 2024.

### Ethical considerations

The Copenhagen ONJ Cohort received ethical approval (protocol no. R-22046553, P-2022–856). All patients provided written informed consent after being informed of treatment alternatives and surgical risks. The study was conducted in accordance with the revised 2024 Declaration of Helsinki [36] and reported in line with the STROBE guidelines [37]. Patient data were anonymised, and no experimental interventions were performed.

### Participants and eligibility criteria

934 patients were diagnosed with MRONJ and included in the cohort. The criteria for diagnosing MRONJ were based on the AAOMS position paper [38] until 2014; after 2014, on the first update [39]; and after 2022, on the latest AAOMS update [2].

Inclusion criteria for the present study were:

- Stage 3 maxillary MRONJ: extensive maxillary defects involving at least half of the maxilla, communication with the maxillary sinus, nasal cavity, or both.
- Surgical reconstruction using a PMTF.

Eligibility was determined based on clinical examination and radiological evaluation using panoramic radiography (OPG), cone beam computed tomography (CBCT), computed tomography (CT), single photon emission computed tomography (SPECT), or combinations thereof.

**Fig. 1:**
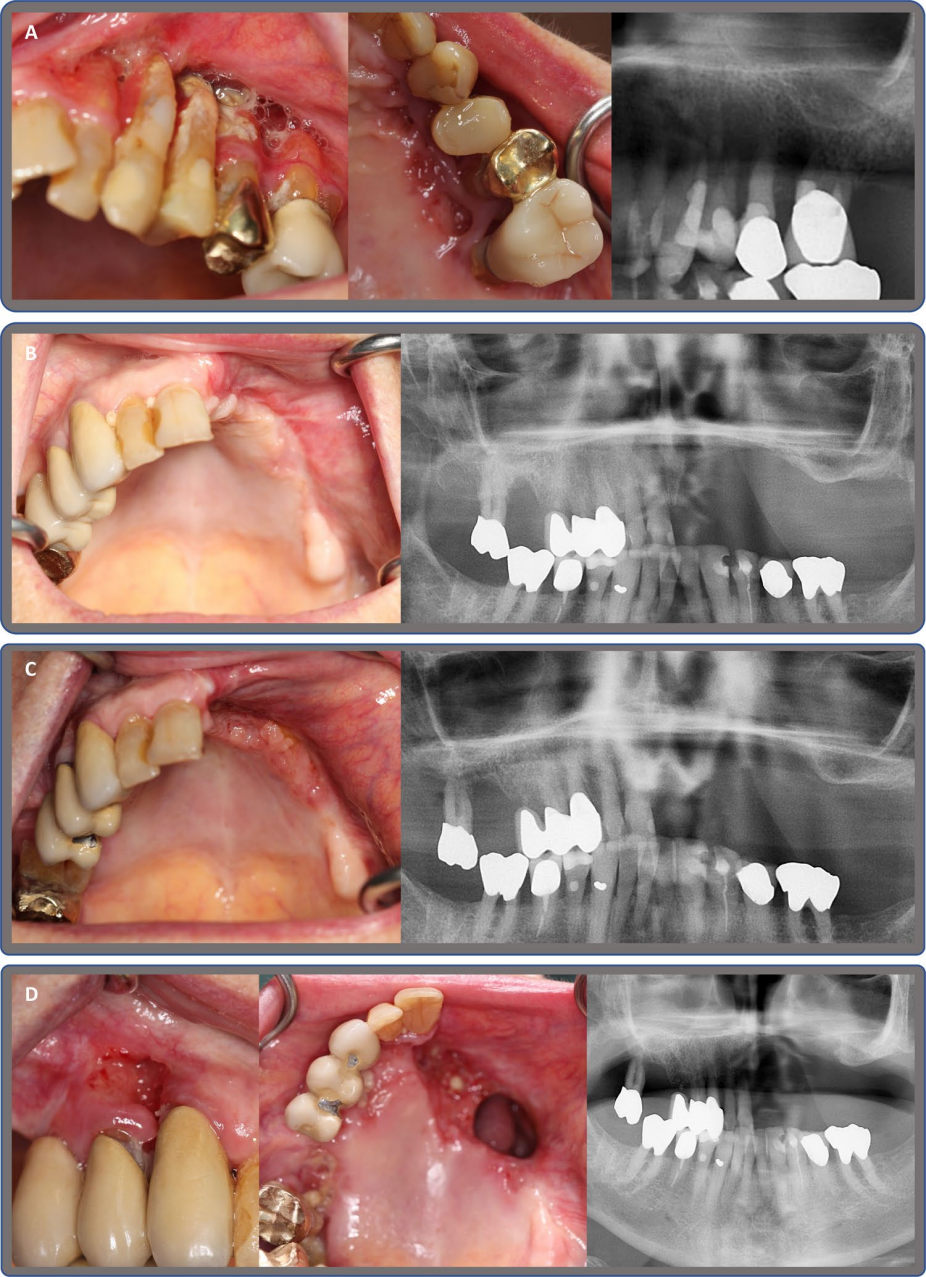

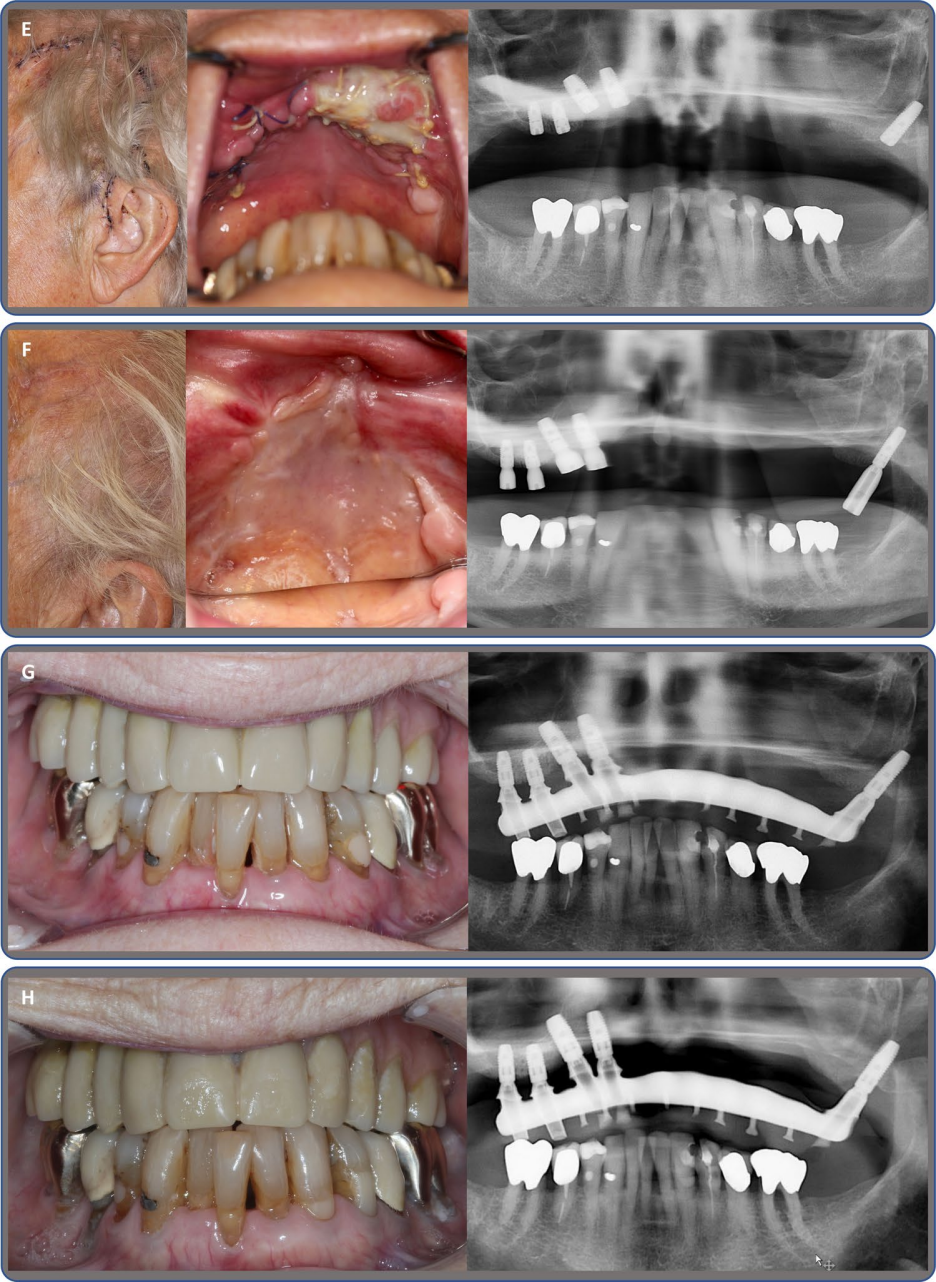
Case 1 presents an 84-year-old female

**Fig. 2:**
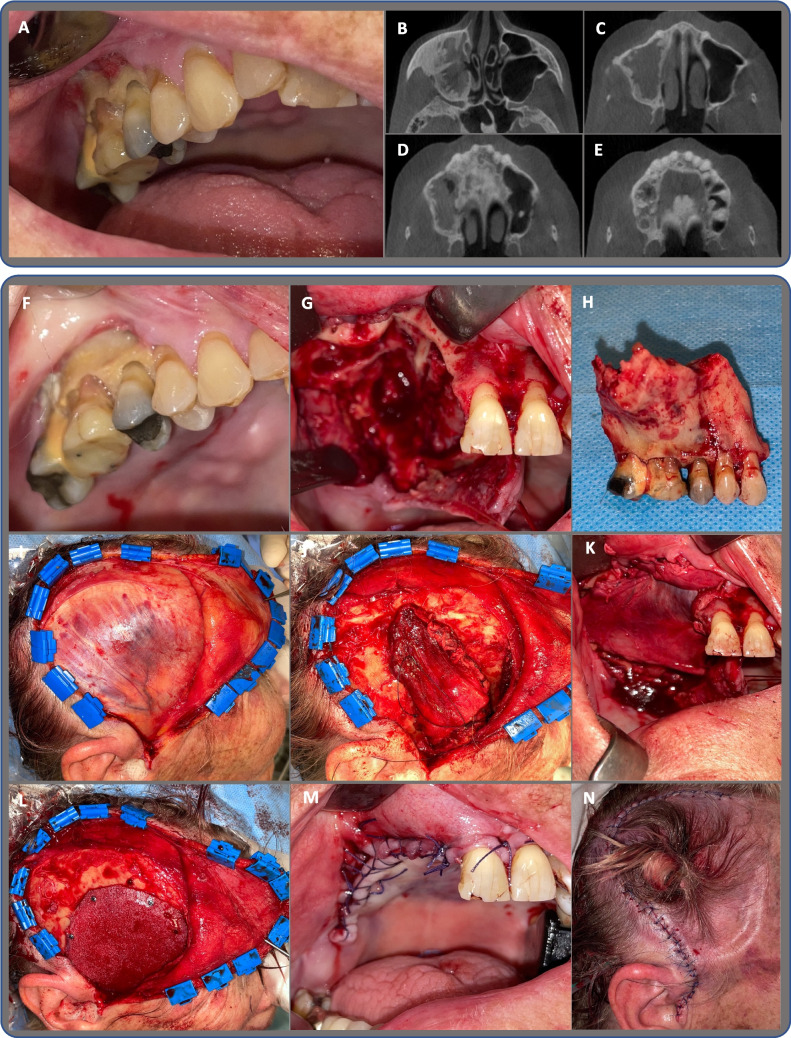

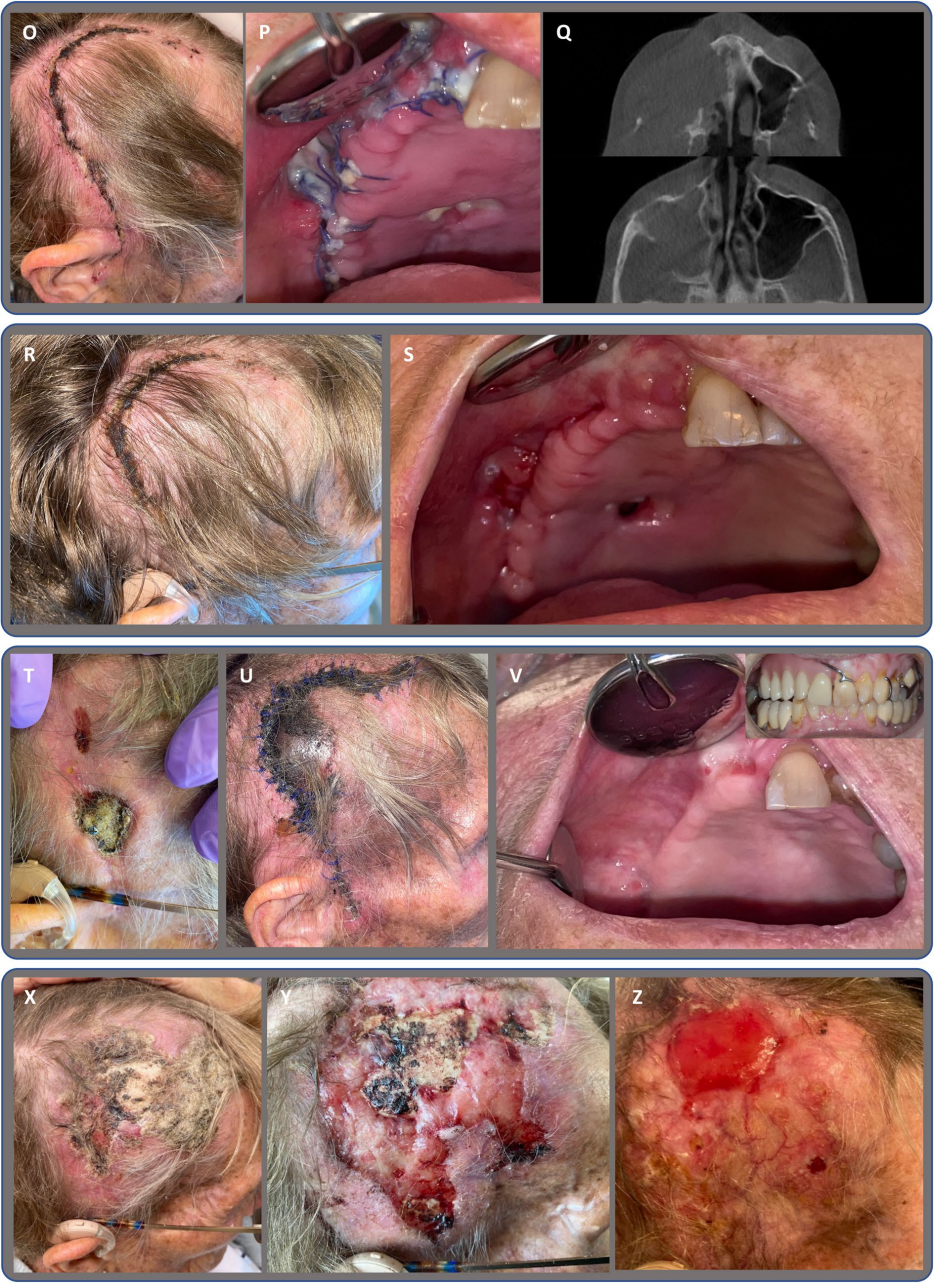
Case 2 presents a 75-year-old female

Although MRONJ is a clinical diagnosis [39], supplementing radiological imaging is needed to evaluate the extent and type of pathology [6, 40, 41]. Routine imaging of patients with MRONJ in our clinic includes a panoramic radiograph (OPG) and a CBCT scan, supplemented with a SPECT/CT scan in cases where there is doubt about the extent of the MRONJ lesion (Fig. 1B, Fig. 2B to 2D). Often, the extent of an MRONJ lesion cannot be properly assessed from an OPG alone [6]. This is also reflected by the fact that maxillary MRONJ lesions tend to be more advanced at the first examination [7, 8].

### Data collection and variables

Demographic data, primary diagnosis, comorbidities, antiresorptive treatment type and duration, smoking status, Eastern Cooperative Oncology Group (ECOG) Performance Status were recorded and pain scores were recorded. Pain was assessed using the Numeric Rating Scale (NRS) and recorded by the operating surgeon during routine preoperative evaluation and at postoperative follow-up visits under standardized clinical conditions. The treatment plan was based on clinical findings, symptoms, radiographic evaluation (OPG, CBCT, CT, or SPECT scans), and the patient's general condition. Radiological imaging was used to assess disease extent and guide surgical planning, as clinical evaluation alone may underestimate maxillary involvement [6–8]. Postoperative CBCT imaging was obtained using a Planmeca ProMax 3D MAX scanner. Defect measurements were conducted in Planmeca Romexis 6 (3D imaging software) using multiplanar reconstructions, and the defect size was recorded as the largest linear dimension measured in the axial plane.

### Surgical procedure

All patients underwent surgery in general anaesthesia supplemented with local anaesthesia (0.5% Marcaine Adrenaline) under sterile conditions. An incision was made on top of the maxillary alveolar process along the exposed bone and the teeth, which had to be removed, with releasing incisions medially and distally as necessary. Necrotic bone was removed, including the teeth involved. Pus, granulation tissue, or both were removed from the maxillary sinuses in conjunction with radical sinusotomy, leaving the sinus without mucosal coverage. Resection of necrotic bone was continued until vital bone was visible clinically. Holes for soft tissue suspension were drilled in the bony edge in the maxillary sinus wall, palatal bone, and through the remaining adjacent alveolar process for later stabilisation of the PMTF with sutures. All excised bone and soft tissue were sent for histopathological examination.

The PMTF was raised through a preauricular approach with anterior temporal extension. After careful dissection and mobilisation, the PMTF was transposed to the oral cavity medially to the zygomatic arch. In neither of the cases was it necessary to remove the coronoid process. The PMTF was sutured (Ethicon Vicryl Suture 3–0, Johnson & Johnson) using the drill holes and the palatal mucosa. The remaining mucoperiosteal flap was mobilised to ensure tension-free closure and sutured partly on top of the PMTF, providing a partial two-layer closure with mattress sutures and interrupted single sutures (Ethicon Vicryl Suture 4–0, Johnson & Johnson). PHDPE implants were customised to the defect and stabilised with osteosynthesis screws to reconstruct the temporal donor site.

### Antibiotic protocol

Patients received amoxicillin/clavulanic acid (500/125 mg) orally three times daily starting one day preoperatively. During hospitalisation (ranging between four and five days), intravenous cefuroxime (1500 mg) was administered three times daily. Postoperatively, oral amoxicillin/clavulanic acid was continued for ten days.

### Follow-up and outcome measures

Patients were followed at 14 days, one month, and at three, six, nine, and twelve months postoperatively, and annually thereafter. Outcomes included intraoral and extraoral healing, signs of infection, donor-site morbidity, facial nerve function, mouth opening, temporal hollowing, and pain using the NRS.

### Statistical analysis

Descriptive statistical analyses were performed due to the low number of patients and the explorative nature of the study.

## Results

### Patient characteristics

From the unpublished Copenhagen ONJ Cohort (*n* = 934; 1 January 2005 to 31 December 2024), maxillary involvement was identified in 29.1% of patients, mandibular involvement in 64.9%, and combined involvement in 6.0%. Twelve patients with stage 3 maxillary MRONJ met the inclusion criteria for reconstruction with a pedicled myofascial temporalis flap (PMTF). Five patients were excluded because they were medically unfit for surgery (*n* = 2) or declined operative treatment in favour of conservative management (*n* = 3). Consequently, seven patients underwent PMTF reconstruction and were included in the analysis.

All patients were female, with a mean age of 77.5 ± 6.3 years. Indications for antiresorptive therapy included malignancy, osteoporosis, or both. Patient demographics, comorbidities, and treatment characteristics are summarised in Table 1.

**Table 1:** Demographic, clinical, and treatment characteristics of patients undergoing pedicled myofascial temporalis flap reconstruction for extensive stage 3 maxillary MRONJ (*n* = 7)

Characteristics	Patient (*n* = 7)
Age, years—mean ± SD (range)	77.5 ± 6.3 (67–84)
Preoperative weight, kg – mean ± SD (range)	56.8 ± 13.8 (41–74.9)
Gender *n* (%)	
Women	7 (100%)
Men	0
ECOG Performance Status, *n* (%)	
0	1 (14.3%)
1	2 (28.6%)
2	0
3	0
Missing data	4 (57.1)
General diagnoses, *n* (%)	
Breast cancer	1 (14.3%)
Osteoporosis	2 (28.6%)
Multiple myeloma	1 (14.3%)
Breast cancer and osteoporosis	3 (42.9%)
Antiresorptive treatment, *n* (%)	
Bisphosphonates	5 (71.4%)
Denosumab	2 (28.6%)
Duration of antiresorptive treatment, months—mean ± SD (range)	
Bisphosphonates	49.9 ± 34.2 (12–96)
Denosumab	120
Antiresorptive treatment stopped, *n* (%)	
Paused antiresorptive before operation	5 (71.4%)
Comorbidity, *n* (%)	
Diabetes	1 (14.3%)
Steroid (1 prior treatment and 3 current treated)	4 (57.1%)
Chemotherapy (1 missing)	5 (71.4%)
Previous or current tobacco user	4 (57.1%)
Dental trauma, *n* (%)	
Tooth extraction before onset of MRONJ	3 (42.6%)
Numeric Rating Scale (NRS) pain from the jaw- mean ± SD (range)	
NRS preoperative (1 missing)	2.0 ± 3.08 (0–5)
NRS postoperative	0
Duration of hospitalisation post-operatively- mean ± SD (range)	4.7 ± 0.5 (4–5)
Follow up, months—mean ± SD (range)	20.3 ± 15.9 (2.2–44.4)
Time from operation to death (4 patients), months – mean ± SD (range)	19.5 ± 19.1 (6–33)

*ECOG Performance Status: 0 = Fully active, able to carry on all pre-disease performance without restriction. 1 = Restricted in physically strenuous activity but ambulatory and able to carry out work of a light or sedentary nature, e.g., light housework, office work. 2 = Ambulatory and capable of all self-care but unable to carry out any work activities. Up and about more than 50% of waking hours. 3 = Capable of only limited self-care, confined to bed or chair for more than 50% of waking hours. 4 = Completely disabled. Cannot carry on self-care. Confined to bed or chair [42]

### Operative findings

Histopathological examination of resected bone confirmed areas of necrosis with appositional bone formation, microbial colonisation, and acute and chronic inflammation in all cases. Resected soft tissues from the maxillary sinus and associated fistulae demonstrated acute and chronic inflammatory changes. No malignant transformation was identified in any specimen.

Postoperative CBCT imaging demonstrated a mean defect size of 1795.0 ± 686.4 mm^2^ (range: 665.0 to 2311.9 mm^2^). Complete closure of all defects was achieved using the PMTF, and no intraoperative complications were recorded.

### Clinical outcomes

All patients achieved uneventful intraoral healing with complete closure of oro-antral and/or oro-nasal communications. No flap-related complications, partial necrosis, or intraoral dehiscence occurred during the follow-up period.

One patient had previously undergone bloc resection with primary closure for maxillary MRONJ and subsequently developed disease recurrence eight months later in an area requiring PMTF reconstruction. Following PMTF reconstruction, stable healing was achieved without further recurrence (case 1).

### Donor-site outcomes

Healing at the temporal donor site was uneventful in six of seven patients. No cases of trismus, permanent facial nerve dysfunction, or clinically significant temporal hollowing were observed. Minimal alopecia along the incision line was noted.

One patient developed a late donor-site complication consisting of wound dehiscence over the PHDPE implant at six months postoperatively, necessitating implant removal. The defect was closed with local flaps. Subsequent wound healing was delayed but ultimately achieved by secondary intention following cessation of systemic corticosteroids and local wound care (case 2). Overall, six of seven PHDPE implants (85.7%) healed without complications.

### Pain and follow-up

The mean preoperative pain score was NRS 2.0 (range 0–5). At one month postoperatively, all patients reported complete pain relief (mean NRS 0).

The mean follow-up period was 20.3 ± 15.9 months (range: 2.2 to 44.4 months). In one patient, the short follow-up duration was due to patient preference and the patients request. Four patients died during the observation period due to underlying disease, with a mean interval of 19.5 ± 19.1 months from surgery to death. No MRONJ-related mortality was observed.

### Case presentation

#### Case 1

An 84-year-old female patient, presented in Fig. 1, was diagnosed with breast cancer in 2009 and osteoporosis in 2011. Comorbidities: hypertension, hypercholesterolemia, chronic renal insufficiency, and uric acid gout. Treated with Alendronate 70 mg weekly for a total of 57 months prior to surgery, with no steroid treatment, but receiving Letrozole treatment due to breast cancer. NRS 2 at the initial visit and spontaneously developed stage 3 MRONJ. A) Clinical photos (facial and palatal) and OPG show stage 3 MRONJ in regions 21, 22, 23, 24, 25, and 26. B) Four months after bloc resection of the region 21 to 27, mucosal healing and no sign of necrosis on OPG. C) Eight months after surgery, clinical pictures show chronic infection in regions 11 to 25. OPG with osteolysis 17, 16, 15 and 11, 21, 22, 23. D) Eleven months after initial surgery, immediately before secondary surgery, planned for maxillectomy, left-side PMTF, right-side buccal fat pad and dental implant operation to achieve later dental rehabilitation. Clinical pictures show chronic infection in the oral mucosa, exposed bone 17, 16, 15 and inferior conchae on the patient's left side. OPG shows osteolytic bone in the alveolar process, bilateral in the maxilla, with the destruction of the sinus and nasal floor and bilateral sinus reaction. E) Fourteen days post-second surgery, healing intra- and extraoral. Intraoral area of the temporal muscle with ongoing epithelisation. OPG shows maxillary resection and dental implants in place. F) Three months after the second surgery, prior to abutment surgery, healing both intra- and extra-orally, with no signs of necrosis. OPG after abutment operation shows signs of osseointegration and no sign of bone loss around dental implants. G) Prosthetic treatment six months after placement of dental implants, Atlantis® Cobalt Chrome acrylic bridge, OPG shows bridge in place, minor bone loss around dental implant 13. No sign of reaction in the right maxillary sinus. H) Three-year follow-up after prosthetic treatment, 3.5 years after the secondary surgery. Dental bridge in situ, no sign of intra- or extraoral infection, no necrotic bone, no pain or discomfort. OPG shows unchanged bone loss at dental implant 13 and only minor bone loss at dental implant 28, with no reaction in the maxillary sinus.

#### Case 2

A 75-year-old female patient, presented in Fig. 2, with multiple myeloma received high-dose anti-resorptive treatment with Denosumab 120 mg every four weeks for 63 months prior to surgery. Simultaneously treated with steroids and chemotherapy, Lenalidomide. No pain. A) The clinical photo shows spontaneous stage 3 MRONJ that has developed in regions 17, 16, 15, and 14. B, C, D, E) CBCT shows periosteal reaction in the right maxillary sinus, sinusitis, osteolytic and osteosclerotic reaction in alveolar process in region of 17, 16, 15, 14, 13, right side in palatal, maxillary and zygomatic bone, with minor sequestrum formation. F) Clinically exposed bone on the day of surgery. G) Following the resection of bone and the removal of granulation tissue, the right side of the nasal and sinus cavities is exposed. H) Resected maxilla, Brown and Shaw classification IIb. I) Temporal muscle after incision. J) Elevation of the temporal muscle, sutures placed in lateral and medial fascia and used to pull the temporal muscle orally on the medial aspect of the zygomatic arch. K) Temporal muscle sutured to bony edges and remaining palatal mucosa. L) PHDPE implants in place fixed with osteosynthesis screws. M) Intraoral sutures. N) Extraoral sutures. O, P, Q) Follow-up 14 days post-surgery, extraoral after suture removal, intraoral and CBCT. R, S) Follow-up 30 days, extraoral healing, intraoral after suture removal. T) Spontaneously developed dehiscence at 3 months follow-up, exposed PHDPE implant. U) Follow-up three weeks after removal of PHDPE implant: extraoral, ischemic necrotic cutis. V) Six-month follow-up intraorally, tooth 11 removed one month prior, and removable prosthesis in place. X) Five months after removal of PHDPE implant – demarcation of necrotic skin and exposed calvaria bone, no infection, paused in dexamethasone. Y) One month after termination of dexamethasone, granulation of cutaneous edges, but unchanged exposed calvaria bone. Z) Three months after referral to plastic surgeons, treated in the special unit of wound care, treated by discontinuation of dexamethasone and local treatment with methylrosaniline at the wound edges, zinc ointment, and application of Synalar. Treatment resulted in hypergranulation over the formerly exposed bone.

## Discussion

This retrospective cohort study evaluated outcomes following reconstruction of extensive stage 3 maxillary MRONJ defects using the PMTF. To the authors’ knowledge, this represents the first published series describing the use of PMTF for this indication and the largest single-centre experience to date. Although maxillary involvement was common within the Copenhagen ONJ Cohort, only a small subset of patients required reconstruction with PMTF, highlighting both the rarity of this approach and the need for individualised treatment planning in advanced disease. The results contributed valuable clinical insights into an uncommon but potentially effective reconstructive strategy for severe stage 3 maxillary MRONJ cases.

All patients in this series achieved successful intraoral healing following radical debridement and tension-free closure with the PMTF. These results reinforce core surgical principles in MRONJ management: complete removal of necrotic bone and robust soft-tissue closure to achieve a watertight seal [13]. The consistency of postoperative success underscores the importance of multilayered reconstruction in advanced maxillary MRONJ defects.

Reconstruction of advanced maxillary MRONJ remains particularly challenging due to compromised vascularity, comorbidities, and the functional and aesthetic demands of the midface [43]. While obturator prostheses remain effective for selected patients [44–46] [19, 47], their success depends on adequate dentition, prosthesis stability, patient dexterity, daily adherence to prosthesis hygiene, and remaining communication [45, 46]. In extensive or unstable defects (particularly in edentulous patients), surgical reconstruction may provide superior functional outcomes [45, 48]. PMTF provides immediate closure of oral-nasal and oral-antral communications, does not require teeth like the obturator for reconstruction, provides a stable base for the prosthesis, and following epithelialisation, the mucosa resembles the adjacent oral mucosa. Additionally, in combination with dental implants, a dental implant-retained prosthesis will restore occlusion and function [45]. All patients were given the option of surgery or a conservative alternative. Consequently, five patients chose conservative treatment due to their health status or preference, while seven patients underwent reconstruction with PMTF. In the present series, PMTF reconstruction enabled durable closure and meaningful pain reduction, with a mean postoperative NRS of 0.

The reconstructive ladder for maxillary MRONJ must be tailored to defect size and patient condition [19, 43, 49]. While local mucoperiosteal flaps are first line, they often fail in large defects due to tension, leading to recurrence [20, 50]. Success in advanced cases hinges on multi-layered, tension-free closure [17, 51]. Although nasolabial flaps offer better closure than mucoperiosteal options [17, 52], their bulk can hinder dental rehabilitation and cause aesthetic complications such as facial scarring and hair growth [53]. The use of the buccal fat pad flap underscores the success of this multiple-layer closure technique, as it has been the workhorse of numerous successfully treated small to medium-sized MRONJ maxillary defect closures in our department [16]. It is a simple, time-efficient technique that epithelialises within three to four weeks. However, it is limited to defects under 50 mm and cannot provide the volume required for extensive reconstruction or palatal midline extension [16, 54]. The defect size in this study ranged from 665 mm^2^ up to 2311.92 mm^2^, with a mean of 1795.03 mm^2^ ± 686.40 mm^2^. This extends beyond the ability of the buccal fat pad flap, particularly when a defect requires additional volume for improved prosthetic rehabilitation. For this reason, the PMTF was utilised for reconstruction.

The PMTF has not been described for the closure of defects related to MRONJ, but it has been successful for other reconstruction purposes [18, 22, 55]. The PMTF is an ideal option for reconstructing medium to large-sized intraoral defects due to its anatomical proximity, vascularity, and adequate bulk. With intraoral re-epithelisation expected within four to six weeks [22, 56]. The findings of this study suggest that the PMTF is a robust alternative for medium-to-large maxillary defects where local flaps are insufficient [22]. Unlike nasolabial flaps, the PMTF re-epithelialises without hair growth concerns and provides the necessary vascularity and bulk for stable closure [22, 56]. In Case 1, PMTF successfully resolved a recurrence following a failed mucoperiosteal flap, maintaining stability and zero pain (NRS = 0) at a three-year follow-up. This highlights the flap's efficacy in managing advanced, recalcitrant MRONJ lesions.

As with any kind of surgical reconstruction, PMTF may present potential complications. Spanio di Spilimbergo et al*.* published a case series on complications after 366 PMTF, divided into periods 1978–1993 (195 patients) and 1994–2012 (171 patients), describing total flap necrosis in 1.5% and 1.7%, intraoral dehiscence in 12.8% and 12.2%, permanent paralysis of the frontal branch of the facial nerve in 12.8% and 12.2%, trismus in 46.8% and 48%, and necessity of removal of alloplastic material from donor side in 17.1% and 7.9%, respectively. In the latter period, only PHDPE material was used in this study, emphasising the success of using this type of implant for donor site reconstruction [18]. Six out of seven PHDPE implants (85.7%) in our study healed without complications. Only one patient developed local dehiscence in the temporal region at a six-month follow-up. The PHDPE implant was removed, and the defect was closed with local flaps. The patient again had healing complications, but the defect eventually healed with the help of the special care unit for wound care. This outcome highlights the vulnerability of this patient population and the importance of meticulous postoperative care, nutritional optimisation, and long-term follow-up.

Compared with microvascular free flaps, PMTF reconstruction offers reduced operative time, lower resource requirements, and avoidance of distant donor-site morbidity [18, 55]. These advantages are particularly relevant in MRONJ patients with advanced malignancy, limited life expectancy, or poor general health, for whom the risk–benefit ratio of free flap reconstruction may be unfavourable [13, 20, 57, 58]. The insidious progression of MRONJ, often with minimal pain, contributes to delayed presentation and extensive bone loss. Once defects exceed the reconstructive capacity of regional flaps, free tissue transfer remains the only option.

Ultimately, our results demonstrate a 100% success rate in treating stage 3 MRONJ-associated defects with the PMTF, with a reduction in mean pain, and no complications related to the flap procedure; however, one patient had a complication related to the donor site reconstruction. There is a constant need to identify risk factors and establish the best possible operating regime, including assessing which patients to operate on and which to exclude. Future investigations should focus on long-term functional outcomes, risk stratification, and optimisation of perioperative protocols to further improve treatment strategies in this challenging patient population. By providing a watertight seal and the potential for dental implant-supported rehabilitation, the PMTF improves HRQoL. Given the insidious progression of MRONJ, which often leads to late presentation and extensive bone loss, the PMTF serves as a vital tool in the reconstructive ladder, balancing surgical risk against the profound benefits of pain relief and functional restoration.

## Limitations

This study has several important limitations. First, its retrospective design led to incomplete and inconsistently documented data, which may have constrained the depth of analysis and introduced information bias. Second, the small sample size markedly limits the generalisability of the findings and reduces the statistical robustness of the conclusions. However, the scarcity of such cases is a positive clinical trend, attributable to improved prevention measures and earlier surgical intervention for MRONJ. Although the number of patients necessitating this particular procedure is limited, the findings remain significant, providing a valuable addition to the surgical literature concerning the management of advanced maxillary MRONJ defects using the PMTF. Third, many of the outcome measures were based on subjective assessments and routine clinical observations rather than validated instruments or standardised evaluation scales, potentially affecting measurement accuracy and comparability. While the results provide useful insights, the limited number of patients and the nature of the available data should be taken into account when interpreting the reliability of the findings and the strength of the conclusions.

## Conclusion

This study suggests that using PMTF for the closure of large oro-antral defects, oro-nasal defects, or both may be a reliable approach for managing MRONJ lesions, with favourable outcomes observed in this cohort. Temporal defect reconstruction following PMTF transposition can be achieved using a PHDPE temporal implant.

## Data Availability

Not applicable.
